# Facing Diseases Caused by Trypanosomatid Parasites: Rational Design of Pd and Pt Complexes With Bioactive Ligands

**DOI:** 10.3389/fchem.2021.816266

**Published:** 2022-01-07

**Authors:** Dinorah Gambino, Lucía Otero

**Affiliations:** Área Química Inorgánica, DEC, Facultad de Química, Universidad de la República, Montevideo, Uruguay

**Keywords:** antiparasitic agents, rational design, trypanosomatid parasites, palladium, platinum

## Abstract

Human African Trypanosomiasis (HAT), Chagas disease or American Trypanosomiasis (CD), and leishmaniases are protozoan infections produced by trypanosomatid parasites belonging to the kinetoplastid order and they constitute an urgent global health problem. In fact, there is an urgent need of more efficient and less toxic chemotherapy for these diseases. Medicinal inorganic chemistry currently offers an attractive option for the rational design of new drugs and, in particular, antiparasitic ones. In this sense, one of the main strategies for the design of metal-based antiparasitic compounds has been the coordination of an organic ligand with known or potential biological activity, to a metal centre or an organometallic *core*. Classical metal coordination complexes or organometallic compounds could be designed as multifunctional agents joining, in a single molecule, different chemical species that could affect different parasitic targets. This review is focused on the rational design of palladium(II) and platinum(II) compounds with bioactive ligands as prospective drugs against trypanosomatid parasites that has been conducted by our group during the last 20 years.

## Introduction

Seventeen infectious diseases have been named by the World Health Organization (WHO) as Neglected Tropical Diseases (NTDs), mainly due to low pharmaceutical industry investment in drug research because of the low associated revenue. Consequently, most of the efforts related to the search of new chemotherapeutics against these diseases has come from the academy. More than 20% of the world’s population lives in regions where these diseases are endemic. The impact of NTDs on the affected countries is enormous because of mortality and morbidity that causes important economic losses (World Health Organization, 2012; De Rycker et al., 2018; Chami and Bundy, 2019; World Health Organization, 2021a).

Human African Trypanosomiasis (HAT), Chagas disease or American Trypanosomiasis (CD), and leishmaniases are protozoan infections produced by trypanosomatid parasites belonging to the kinetoplastid order. These are among the most important neglected diseases and constitute an urgent health problem in developing countries. They are often co-endemic in certain regions of the world (Leishmaniasis and Chagas’ disease in South America and Leishmaniasis and HAT in Africa) and they have span worldwide because of globalization caused by human migration. Despite being some of the most life-threatening infective diseases, only a poor and inadequate chemotherapy is available (De Rycker et al., 2018; Santos et al., 2020; Kourbeli et al., 2021).

American trypanosomiasis also called Chagas’ disease after its discoverer, the Brazilian scientist Carlos Chagas, is endemic in Latin America where there are 7–8 million infected people, 10,000 annual deaths and 25 million people at risk of infection. In the last decades, the number of cases in non-endemic regions, like United States, Australia, Europe and Japan, has increased due to migration flows. The disease is caused by the protozoan parasite *Trypanosoma cruzi* (*T. cruzi*) that is mainly transmitted to mammalian hosts by infected blood-sucking bugs. In addition, other modes of transmission, responsible of the spreading of the disease to non-endemic regions, involve blood transfusion, organ transplant and congenital transmission. The parasite shows a complex life cycle that involves stages in the host and in the biological vector. Stages in the host show different susceptibility to drugs, which hampers the development of an effective chemotherapy (Santos et al., 2020; World Health Organization, nd).

**FIGURE 1:**
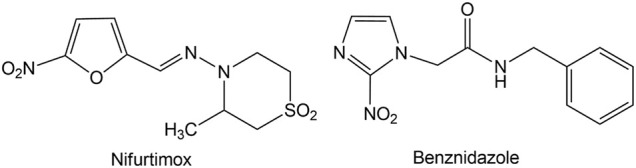
Structure of Nifurtimox and Benznidazole.

The available chemotherapy includes drugs developed more than 50 years ago, Benznidazole and Nifurtimox (Figure 1), which proved to be toxic, require long treatments, are not effective in chronic stage of the disease, and often develop resistance.

Many natural products and synthetic inorganic and organic compounds have been successful assayed against *T. cruzi* and some of them entered clinical trials but finally did not reach the clinics (Paucar et al., 2016; Chatelain and Ioset, 2018; Scarim et al., 2018; Francisco et al., 2020).

**FIGURE 2:**
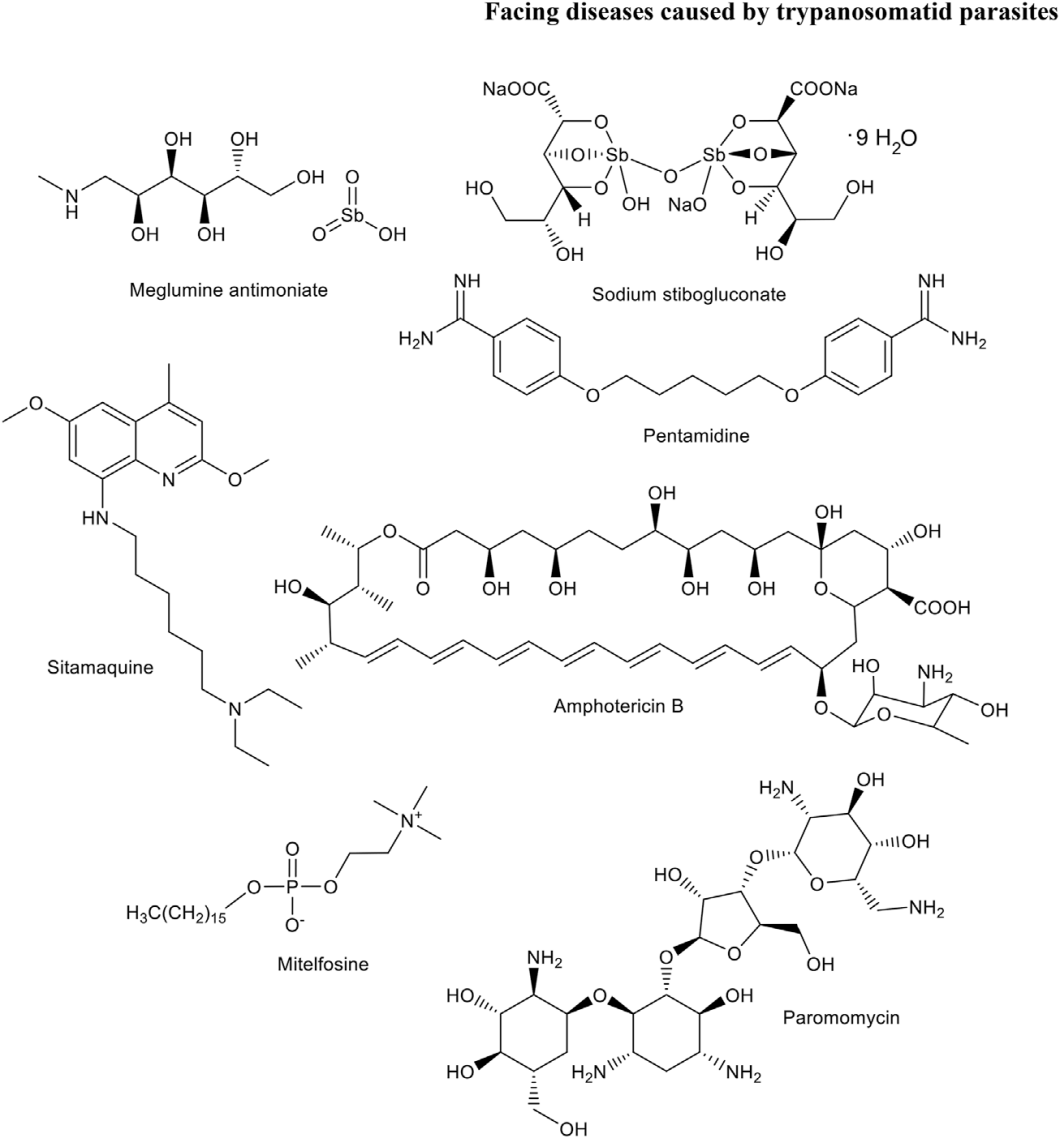
Some current drugs for the treatment of Leishmaniasis.

Leishmaniases are a group of diseases caused by various *Leishmania* species. The insect vector that transmits the disease through its bite is a female phlebotomine sandfly. Leishmaniasis affect 350 million people in 98 countries, in four continents. There are three main forms of the disease: cutaneous, visceral and mucocutaneous with different levels of severity. In addition, Leishmania–HIV co-infection has been reported leading to more severe forms that are also more difficult to face (Nagle et al., 2014; Burza et al., 2018; Kwofie et al., 2020; Brindha et al., 2021; World Health Organization, 2021b). Some current drugs for the treatment of this disease are shown in Figure 2.

**FIGURE 3:**
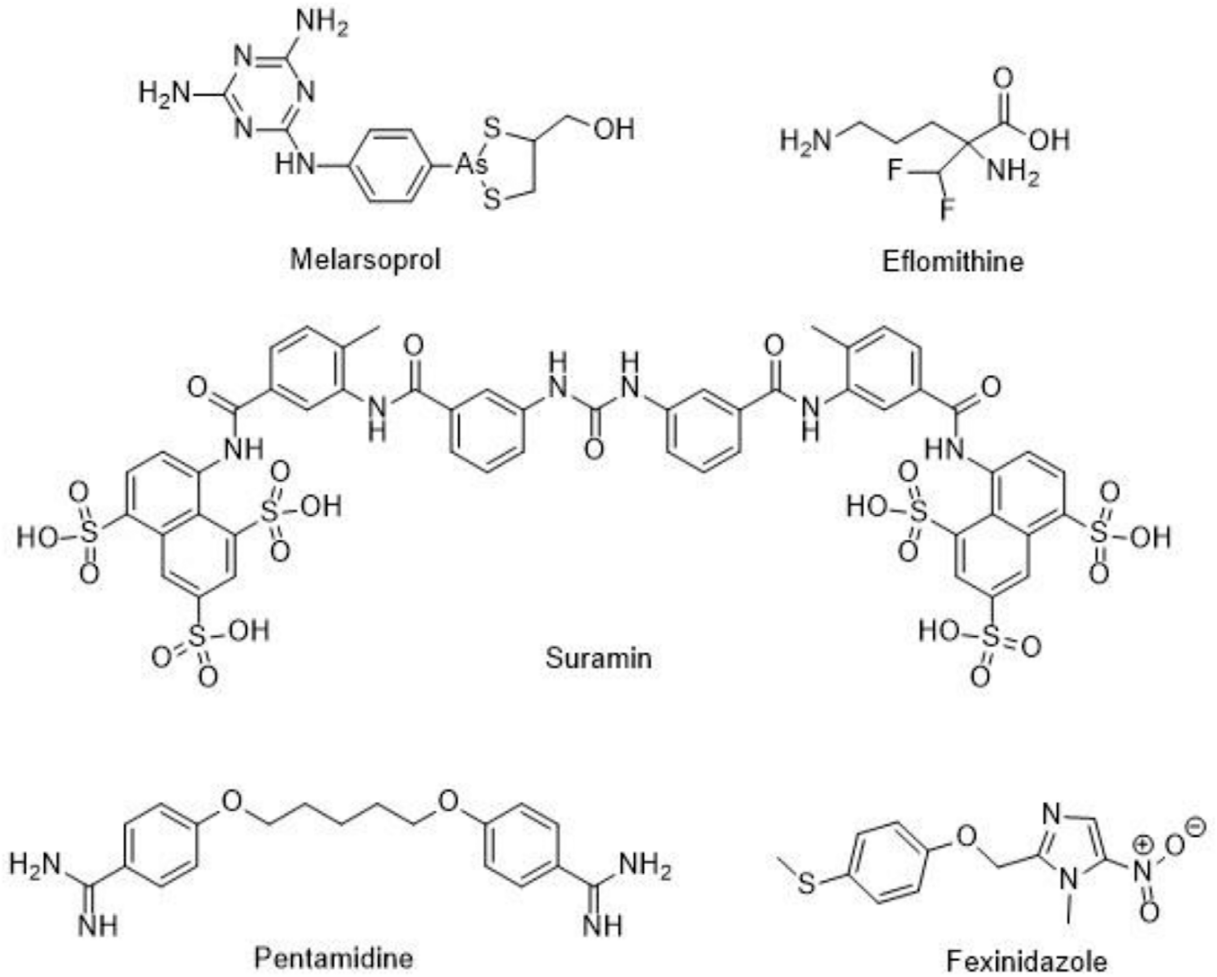
Drugs for the treatment of HAT.

HAT mainly occurs in the sub-Saharan regions of Africa and it is transmitted through the bite of a tsetse fly. During the 20th century, the epidemics of this disease was devasting being people living in rural areas the most affected ones. Despite control efforts have reduced the number of annual cases, the relax of surveillance policies, the lack of new drugs for its treatment and the emergence of resistance to the old ones, have contributed to the reappearance of the disease. The protozoan parasite causing this disease is *Trypanosoma brucei* (*T. brucei*) and in particular, *Trypanosoma brucei gambiense* and *Trypanosoma brucei rhodesiense* subspecies. Most cases of chronic-like infections are caused by *T. b. gambiense* while *T. b. rhodesiense* causes the most serious form of the disease. In the late stage of this illness the parasites enter the central nervous system leading to profound sleep effects that give rise to the common name of HAT, sleeping sickness. A similar disease, called Nagana importantly affects cattle since antiquity but, interestingly, humans are resistant to the trypanosome species causing it. All forms of HAT are fatal if they are not properly treated. Five drugs are currently recommended: pentamidine, melarsoprol, eflornithine, suramin, and fexinidazole (Figure 3). Nifurtimox-eflornithine combination therapy is currently a first line treatment for HAT.

However, most of these drugs show toxicity problems and their efficacy is variable depending on the type and stage of the disease. Although the mortality rates for HAT have decreased substantially in the last years with less than 1,000 cases found in 2019, the development of improved drugs bearing high bioavailability and low toxicity is crucial to definitively fight HAT (Nagle et al., 2014; Kourbeli et al., 2021; World Health Organization, 2021c).

Even though in the last years public-private consortia have pushed forward the drug discovery process, in general, no new drugs to treat diseases caused by kinetoplastids have been registered in more than 30 years. As previously stated, current chemotherapy against diseases caused by trypanosomatid parasites is very deficient so more research is urgently needed to discover safe and effective therapies (Nagle et al., 2014; Rao et al., 2019; Kayode et al., 2020).

During the preclinical development of new prospective drugs, it is important to know that these parasites exhibit complicated lifecycles alternating between stages in the insect vectors gut and in the mammalian host. This knowledge is relevant since it is well known that these different stages show different biological properties and, most importantly, different susceptibility to drugs. Shortly, *T. cruzi* is transmitted to the vertebrate host by the release of metacyclic trypomastigote form of the parasite in the feces of an infected triatome insect vector during its bite. Once inside the host, the parasite converts into the infective blood circulating trypomastigote stage that invades the tissues. Intracellularly, the amastigote form emerges that divides by binary fission and produces cell´s lysis. The released amastigotes convert into blood circulating trypomastigotes. After an insect meal they infect new insects and close the life cycle by converting into the non-infective epimastigote stage that only exists inside the gut of the insect. A similar scenario is observed in the lifecycle of *T*. *brucei* that shows four main developmental stages that occur in the infected insect and in the mammalian host, epimastigotes and procyclic forms, slender metacyclic trypomastigotes, and stumpy bloodstream proliferative metacyclic trypomastigotes, being the last one the most relevant form in the development of therapeutic agents. In contrast to other trypanosomatids, the part of the life cycle of *T*. *brucei* in the mammalian host occurs completely in the extracellular space. During the lifecycle of *Leishmania sp* the parasite alternates between the infective promastigote stage generated in the intestine of the insect vector and the replicative amastigote form inside the host macrophages (Brindha et al., 2021; Kourbeli et al., 2021).

Although these trypanosomatid protozoan parasites show important differences, they are transmitted by different insect vectors and are responsible for clinically different human diseases, it has been demonstrated that they show relevant similarities related to their biology. Their genomes have been sequenced in 2005 and they are available in a database for parasites of the family *Trypanosomatidae* (TriTrypDB, http://tritrypdb.org) (El-Sayed et al., 2005; Aslett et al., 2010). These studies showed that *Leishmania* spp, *T. cruzi* and *Trypanosoma brucei* have many common features, like gene conservation, genome architecture, high synteny, identical amino acid sequences in proteins and common subcellular structures like kinetoplasts. In particular, they show more than 6,100 closely related genes codifying proteins of a total of 8,000–12,000 genes. This paves the way for planning the development of broad-spectrum compounds that could be trypanosomatid-specific, affecting the three main parasites instead of affecting only one of them. This could lead to drugs that affect common targets in the different parasites showing activity against *Leishmania* spp, *T. cruzi* and *T. brucei* (Stuart et al., 2008; Ilari et al., 2018).

Metal-based medicines have been used since ancient times. However, the discovery of the potent anti-tumor activity of cisplatin, [PtCl_2_(NH_3_)_2_] determined the beginning of the modern era of Medicinal inorganic chemistry, accompanied by a huge impact of metal compounds in modern medicine. By exploiting the unique properties of metal ions, Medicinal inorganic chemistry currently offers an attractive option for the rational design of new drugs, looking for defined targets and activities, and new diagnostic and theranostic tools (Zhang and Sadler, 2017; Hanif and Hartinger, 2018; Englinger et al., 2019; Kostelnik and Orvig, 2019; Soldevila-Barreda and Metzler-Nolte, 2019; WahsnerGale et al., 2019; Wang et al., 2019; Anthony et al., 2020; Chamberlain et al., 2020; Chellan and Sadler, 2020; Imberti et al., 2020; Murray and Dyson, 2020; Cirri et al., 2021; Karges and Cohen, 2021; Lin et al., 2021; Silva et al., 2021; Yousuf et al., 2021). In the last decades, the development of metal-based drugs has proven to be a promising approach in the search for new therapeutic tools against parasitic diseases. As a result of the research performed by several academic groups, a great number of classical coordination compounds and organometallics bearing antiparasitic activity were developed (Sánchez-Delgado et al., 2004; Navarro, 2009; Navarro et al., 2010; Gambino, 2011; Gambino and Otero, 2012; Salas et al., 2013; Tahghighi, 2014; Brown and Hyland, 2015; Pessoa et al., 2015; Camarada et al., 2016; Gambino and Otero, 2018; Marcelino et al., 2018; Ravera et al., 2018; Gambino and Otero, 2019; Markwalter et al., 2019; Ong et al., 2019; Mbaba et al., 2020; Păunescu et al., 2021).

## Strategies for the Rational Design of Antiparasitic Metal Complexes

As previously stated, current drugs for the treatment of diseases caused by trypanosomatid parasites are characterized for their severe side effects, the need for prolonged treatments, and the emergence of resistance. In the search for new improved treatment options based on organic compounds, both phenotypic and target-based approaches have been used. In a phenotypic approach, many compounds are evaluated *in vitro* against the parasites searching for a hit or lead compound and, in the target-based drug design, a specific parasite target is selected, and drugs are drawn up to affect this target specifically (Haanstra and Bakker, 2015; Field et al., 2017; Gambino and Otero, 2019).

The *phenotypic strategy* has been the method of choice for the discovery of potential metal-based drugs and, in particular, antiparasitic ones. In fact, although metal compounds have the potentiality of interacting with selected parasitic enzymes and biomolecules, they are likely to act on different targets at the same time. In addition, they are usually transformed *in vivo* into active species by ligand exchange or redox reactions which sometimes makes it difficult to design them to act on specific targets (Navarro et al., 2010; Field et al., 2017; Anthony et al., 2020).

However, metal-based classical coordination complexes or organometallic compounds could be designed as multifunctional agents joining, in a single molecule, different chemical species that could affect different parasitic targets: a bioactive metal ion or metal centre and one or more antiparasitic organic compounds included as ligands. In fact, metal complexes with compounds bearing antiparasitic activity as ligands are expected to maintain the target of the bioactive compound. In addition, the presence of some bioactive metal ions (like palladium or platinum, as will be stated below) could give rise to the appearance of other targets like DNA or parasitic enzymes (Sánchez-Delgado et al., 2004; Gambino, 2011; Gambino and Otero, 2012; Gambino and Otero, 2018; Gambino and Otero, 2019).

In this sense, the peculiar chemical properties of metal complexes impart them the ability to interact with different biomolecules including proteins, enzymes, small peptides, nucleic acids, carbohydrates, among many others. To avoid unspecific toxicity, the design of metal-based compounds bearing antiparasitic activity should include specific targets like proteins or enzymes that are essential for the parasites and are not present in the host (like NADH-fumarate reductase, see below). When the mode of action of metal compounds are involved in metabolic pathways or targets that are common between parasite and mammals, specificity in the toxic action could also be achieved by taking advantage of other differences like the presence of specific organelles in the parasites or the poor mechanisms of detoxification of some toxic species as will be stated in the following sections (Gambino and Otero, 2019).

In particular, DNA is not a specific parasitic target as it is for metal-based antitumor compounds. However, parasites and cancer cells have some common features like their capacity for rapid cell division, some immune evasion and defense strategies, high rate of aerobic glycolysis with key glycolytic enzymes, need of nucleic acid biosynthesis, etc. These similarities noy only support DNA as a valid target for antiparasitic metal-based drugs but also the selection of metal centres that have proved to be useful in cancer therapy, like platinum or palladium (see below) (Williamson and Scott-Finnigan, 1975; Kinnamon et al., 1979; Fuertes et al., 2008; Dorosti et al., 2014; Sullivan et al., 2015).

In the framework of these general features, one of the main strategies for the design of metal-based antiparasitic compounds has been the coordination of an organic ligand with known or potential biological activity, to a metal centre or an organometallic *core*. The activity of the selected ligand could be enhanced because of metal complexation and its pharmacological properties could be improved as the stabilization of the organic drug may allow it to have a better performance in reaching and affecting the biological targets. In addition, the potential toxicity of the metal may be reduced because the organic ligand may limit the ability of the metal to interact with biomolecules that leads to toxicity. This approach could also be useful to circumvent drug resistance, as the metal complex would mask the organic drug. Finally, as stated, these metallodrugs would be capable of affecting multiple parasitic targets simultaneously (Sánchez-Delgado et al., 2004; Gambino and Otero, 2012; Ong et al., 2019; Navarro and Visbal, 2021).

For a metal compound to be able to accomplish an adequate pharmacological behavior, a very strict control of all its chemical features should be considered. The selection of the nature and oxidation state of the metal, the nature and number of coordinated ligands and the coordination geometry are essential for controlling complexes’ reactivity and tuning their thermodynamic and kinetic stability. In addition, other physicochemical properties like lipophilicity, solubility or protein interaction can also be tuned by the choice of the metal ion, its oxidation state, and the inclusion of auxiliary co-ligands (Barry and Sadler, 2014; Ong et al., 2019; Czarnomysy et al., 2021; Mjos and Orvig, 2014).

This review is focused on the rational design of palladium(II) and platinum(II) compounds with bioactive ligands as prospective antiparasitic drugs that has been conducted by our group during the last 20 years. The selection of these metal ions is based on the resemblance between tumour cells and parasites as stated above. In this sense, the current treatment of cancer is based on platinum complexes, alone or in combination with other chemotherapeutic agents. In addition, most efforts related to the development of more effective and less toxic anticancer agents have been devoted to platinum compounds. On the other hand, complexes containing palladium are closely related to their platinum analogues. Therefore, Pd(II) has been selected as an alternative to Pt(II) in the search of new compounds for cancer therapy. The most significant resemblances are related to their coordination chemistry and, in particular, to the coordination number (four) and geometry (square planar) of their compounds. However, the ligand exchange kinetics of Pd(II) compounds is 10^4^–10^5^ times faster than that of the Pt(II) analogues. This means that Pd(II) compounds are much more reactive in solution which could lead to a higher toxicity and a different biological behaviour. However, the adequate selection of ligands could be able to stabilize Pd(II) complexes affecting their reactivity and imparting substitution inertness (Jahromi et al., 2016; Lazarević et al., 2017; Cirri et al., 2021; Czarnomysy et al., 2021; Yousuf et al., 2021).

## Palladium and Platinum Complexes With Bioactive Ligands

**FIGURE 4:**
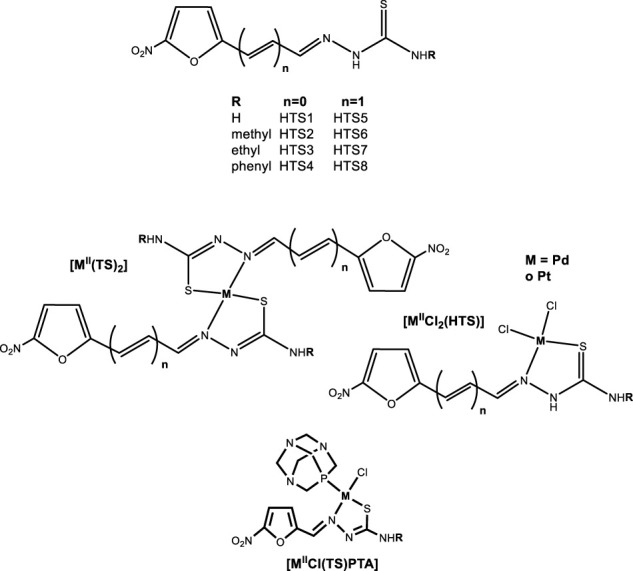
Bioactive 5-nitrofuryl-containing thiosemicarbazones (HTS) and their Pt(II) and Pd(II) complexes.

In the search of ligands bearing anti *T. cruzi* activity, we had developed 5-nitrofuryl-containing thiosemicarbazones (HTS) maintaining the 5-nitrofuryl moiety that has proved to be the pharmacophore group of Nifurtimox (Figure 4). HTS were more active *in vitro* against *T. cruzi* than this reference drug. These compounds were designed to act on *T. cruzi* by the same mechanism than Nifurtimox: the generation of toxic reactive oxygen species (ROS) through the reduction of the nitro moiety followed by redox cycling. In addition, they have proved to affect the activity of trypanothione reductase parasitic specific enzyme through the nitrofuran group as well as to inhibit the main parasitic cysteine protease, cruzipain, through the thiosemicarbazone moiety (Aguirre et al., 2004; Rigol et al., 2005; Otero et al., 2008).

In order to address the effect of palladium and platinum complexation on the anti *T. cruzi* activity of these organic compounds, a large series of complexes with different stoichiometries and including different co-ligands was developed (Figure 4).

Thirty two analogous platinum and palladium complexes of the formula [MCl_2_(HTS)] and [M(TS)_2_] with M = Pd(II) and Pt(II) were obtained. Most of them were*, in vitro*, equally or more active than Nifurtimox against *T. cruzi* (epimastigote form, Tulahuen 2 strain). For palladium complexes, the activity of the ligands was maintained or increased as a consequence of metal complexation. Although, the obtained IC_50_ differences between the assayed species were quite low (IC_50_/5 days between 3 and 6 µM), a general trend was found: [PdCl_2_(HTS)] > HTS > [Pd(TS)_2_]. However, no similar trend was observed for platinum compounds showing, in most cases, lower activity than the palladium counterparts and the free ligands. Some platinum compounds were also tested against Dm28c epimastigotes and trypomastigotes of *T. cruzi*. Indeed, the infective trypomastigote form of the parasite resulted more susceptible to most of the tested Pt compounds than the epimastigote form, being these compounds more active on trypomastigotes than Nifurtimox (Otero et al., 2006; Vieites et al., 2008a; Vieites et al., 2009).

On the other hand, eight new palladium(II) and platinum(II) complexes of formula [MCl(TS)(PTA)] with PTA (1,3,5-triaza-7-phosphaadamantane) as co-ligand were obtained. PTA was included with the aim of modulating the solubility and lipophilicity of the new species. Most [MCl(TS)(PTA)] complexes showed similar activities against *T. cruzi* (trypomastigote form, Dm28c clone) to those of the corresponding HTS ligands and [PtCl_2_(HTS)] complexes. In contrast to what was observed for [MCl_2_(HTS)] compounds, no significant differences between palladium and platinum complexes were observed. However, for the most active compound, [MCl(TS4)(PTA)], the selectivity index (SI) was higher for the platinum complex (SI =20) than for the palladium one (SI = 10). It also should be noted that for the whole series of compounds, no correlation between the anti *T. cruzi* activity and the nature of substituent in the thiosemicarbazone chain was observed (Cipriani et al., 2014).

All prepared palladium and platinum complexes with the 5-nitrofuryl-containing thiosemicarbazones as bioactive ligands were supposed to show various mechanisms of action i.e., affecting the same targets or processes than the bioactive ligands or maintaining those mechanisms related to the PTA co-ligand and/or to the metal ions. Therefore, different studies were performed in order to sustain the proposed targets and to look into the potential mode of action of the metal compounds. Related to the bioactive ligands, the reduction of the nitro moiety had been demonstrated to be the first step of their mechanism of anti *T. cruzi* action. The nitro anion formed would be responsible of generating other toxic radical species through a redox cycling process. Therefore, the effect of metal complexation on the redox potential of the nitro moiety was studied using cyclic voltammetry. This potential slightly changed because of both palladium or platinum complexes formation (Δ*E* = 0.05–0.1 V) and no significant differences were observed between both metal ions. However, it should be stated that the nitro moiety of all compounds resulted more easily reducible than that of Nifurtimox, and, therefore, the capacity of generating toxic free radicals would be better for the complexes. The production of free radicals inside the parasite cells was assessed by ESR spectroscopy using 5,5-dimethyl-1-pirroline-N-oxide (DMPO) for spin trapping of radical species having short half-lives. A 10–13 lines spectral pattern was observed for all the studied complexes which is consistent with the intracellular generation of the hydroxyl radical and the nitroheterocyclic radical of the complexes. So, the mechanism of anti *T. cruzi* action of the HTS compounds seems to remain in the obtained complexes. However, only for [PdCl_2_(HTS)] and [Pd(TS)_2_] series, a good correlation between the concentration of the detected radicals (measured through the EPR signal intensities) and the IC_50_ values for the anti *T. cruzi* activity was observed. On the other hand, the described redox cycling processes should increase the parasite oxygen consumption. Thus, the parasite oxygen uptake in the presence of the compounds was determined. In general, complexes increased oxygen consumption which confirms that redox cycling processes are occurring inside the parasites treated with the complexes (Otero et al., 2006; Vieites et al., 2008a; Cipriani et al., 2014).

On the other hand, trypanothione reductase and cruzipain inhibition was also observed for some of the obtained metal compounds but no correlation with the anti *T. cruzi* activity was observed (Otero et al., 2006; Cipriani et al., 2014).

DNA was also tested as a potential target for the developed palladium and platinum compounds. The binding of [MCl_2_(HTS)] and [M(TS)_2_] compounds was studied by combining quantification of the bound metal by atomic absorption spectrometry and quantification of DNA by electronic absorption measurements. The amount of metal bound to DNA for platinum complexes was comparable to that previously reported for cytotoxic metal complexes and it was lower than the one determined for palladium complexes (Otero et al., 2006; Vieites et al., 2008a; Vieites et al., 2009).

[MCl_2_(HTS)] interaction with DNA was characterized by using gel electrophoresis, DNA viscosity measurements, circular dichroism (CD) and atomic force microscopy (AFM). Electrophoresis results showed that all complexes caused the loss of DNA superhelicity and modifications in the shape of plasmid DNA were observed in AFM studies. The effect on DNA was more significant for palladium complexes than for platinum analogues. In addition, CD results showed that while palladium complexes induce modifications in calf thymus DNA structure, no effect was observed for platinum ones. Finally, either Pd or Pt complexes increased the viscosity of DNA which agrees with an intercalative mode of interaction. An explanation for the observed differential intensity of the effect on DNA between palladium and platinum complexes could be the differences in exchange reactions kinetics between both metal ions (Vieites et al., 2011).

Similar results were obtained for [MCl(TS)(PTA)] complexes (Figure 4). The effect of these compounds on DNA was characterized by gel electrophoresis and ethidium bromide fluorescence experiments. Results of both experiments are in accordance with an intercalating-like mode of interaction between DNA and these compounds. However, the intensity of the effect on DNA was dependent on the nature of the metal ion. In fact, all Pd complexes showed a more significant effect on DNA than the platinum ones. In fact, some palladium compounds induce decomposition of DNA at high DNA/complex molar ratios (Cipriani et al., 2014).

It is interesting to note that no correlation between the anti *T. cruzi* activity and DNA binding was observed for the different series of palladium and platinum compounds. In fact, theoretical calculations performed for [MCl_2_(HTS)] complexes suggested that, in the cell, these complexes would not interact with DNA because they would react with the cell content before accessing to DNA. In addition, the potency and mode of interaction of both HTS and their metal complexes with *T. cruzi* cruzipain and trypanothione reductase enzymes was also studied using molecular docking. Results showed that the mode of action of these compounds involved multiple mechanisms and that, depending on the nature of the species, one mode of action would be predominant over others (Merlino et al., 2011).

**FIGURE 5:**
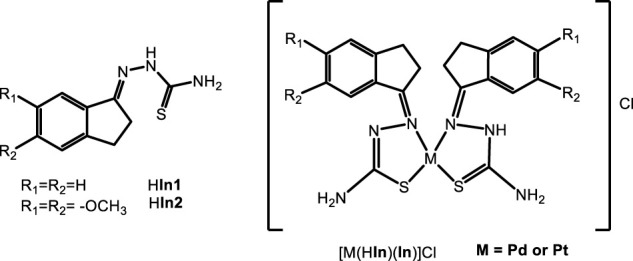
Bioactive thiosemicarbazones derived from 1-indanones (HIn) and their Pt(II) and Pd(II) complexes.

Other thiosemicarbazone containing bioactive ligands were selected to study the effect of palladium and platinum complexation on the anti *T. cruzi* activity. Eight Pd(II) and Pt(II) complexes, [MCl_2_(HIn)] and [M(HIn)(In)]Cl with HIn = thiosemicarbazones derived from 1-indanones were obtained (Figure 5; Gómez et al., 2011).

The *in vitro* activity on *T. cruzi* (epimastigote form, Tulahuen 2 strain) and the unspecific cytotoxicity on red blood cells, were studied. All compounds showed higher activity than the corresponding free ligands with IC_50_ values in the low micromolar range. Most palladium compounds showed higher trypanosomicidal activity than their platinum counterparts. A quite good correlation between lipophilicity and antiproliferative activity was observed for these complexes. On one hand, lipophilicity was enhanced as a consequence of metal complexation and, in most cases, anti *T. cruzi* activity was also increased. In addition, being most palladium complexes more lipophilic than platinum ones, they showed higher anti *T. cruzi* activity. Unfortunately, obtained complexes showed low selectivity for the antiparasitic action, being less selective than the free ligands. In this case, coordination to Pd and Pt led to an increase in bioactivity but had a deleterious effect on unspecific cytotoxicity.

On the other hand, obtained complexes were tested for their antitumoral activity. Free ligands had no cytotoxic effect, but platinum and palladium complexes showed anti-leukemia properties and induced apoptosis. However, in this case no clear correlation between antitrypanosomal and antitumoral activities could be detected (Gómez et al., 2011; Santos et al., 2012).

**FIGURE 6:**
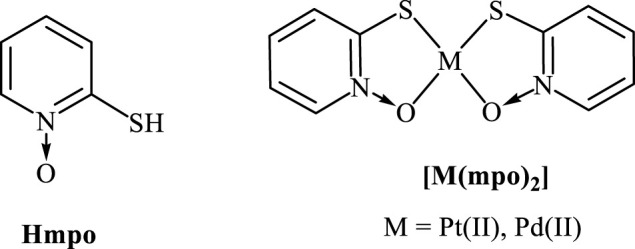
pyridine-2-thiol *N*-oxide (Hmpo) and their Pt(II) and Pd(II) complexes.

Using the same approach, palladium and platinum compounds with pyridine-2-thiol *N*-oxide (2-mercaptopyridine *N*-oxide, Hmpo) as bioactive ligand were studied as potential antitrypanosomal agents (Figure 6). Mpo had shown a high anti *T. cruzi* activity against all forms of the parasite and no unspecific toxicity on mammalian cells. The antiparasitic action of mpo was related to the inhibition of NADH-fumarate reductase enzyme which is responsible for producing succinate from fumarate in the parasite. The lack of this enzyme in mammalian cells makes it a promising target for the development of antichagasic compounds. In addition, mpo, as similar amine *N*-oxides do, could suffer bioreduction leading to the release of radical species that are toxic for the parasite (Vieites et al., 2008b).

[Pd(mpo)_2_] and [Pt(mpo)_2_] compounds were synthesized and fully characterized. Both complexes showed very high *in vitro* growth inhibition activity of *T. cruzi* (epimastigote form, Tulahuen 2 strain) with IC_50_ values in the nanomolar range (IC_50_/5 days = 0.067 and 0.200 µM for palladium and platinum complexes, respectively). They were 39–115 times more active than Nifurtimox. In addition, the palladium complex showed an approximately threefold enhancement of the activity compared with the free mpo while only a low increase in the activity was observed for the platinum compound. In addition, owing to their low unspecific cytotoxicity on mammalian macrophages, the complexes showed a highly selective antiparasitic activity.

These complexes were also good candidates for a multi-target activity. In this sense, free-radical production, inhibition of the parasite-specific enzymes trypanothione reductase and NADH-fumarate reductase were studied. Additionally, studies on DNA interaction were performed.

Although radical species could be obtained electrochemically for both metal complexes, no free radical species were detected when they were incubated with the epimastigote form of *T. cruzi*. In addition, neither trypanothione reductase inhibition nor DNA interaction could be observed.

However, in the assayed conditions, both complexes have an inhibitory effect on NADH fumarate reductase. [Pd(mpo)_2_] showed the highest inhibition levels while the effect of [Pt(mpo)_2_] on the anzyme was similar to that of the free ligand. A similar behaviour was observed when analysing the IC_50_ values of these compounds which strongly suggest the involvement of this enzyme in the mode of action of the obtained complexes.

On the other hand, homology modelling combined with enzyme-cofactor docking were used to propose tertiary structures for NADH-dependent *T. cruzi* fumarate reductase. This model was used to explain the inhibitory effect and the binding modes of Pd- and Pt-mpo complexes. In fact, obtained theoretical inhibition constants (*K*_i_ values) showed a good correlation with the experimental data. Both complexes bind to the enzyme into the cleft between domains 2 and 3, near to the nicotinamide ring of the NADH cofactor. However, [Pd(mpo)_2_] seems to bind closer to the cofactor which could affect the orientation of the nicotinamide ring for a proper position for catalysis, explaining the better inhibitory capacity displayed by this complex (Merlino et al., 2014).

**FIGURE 7:**
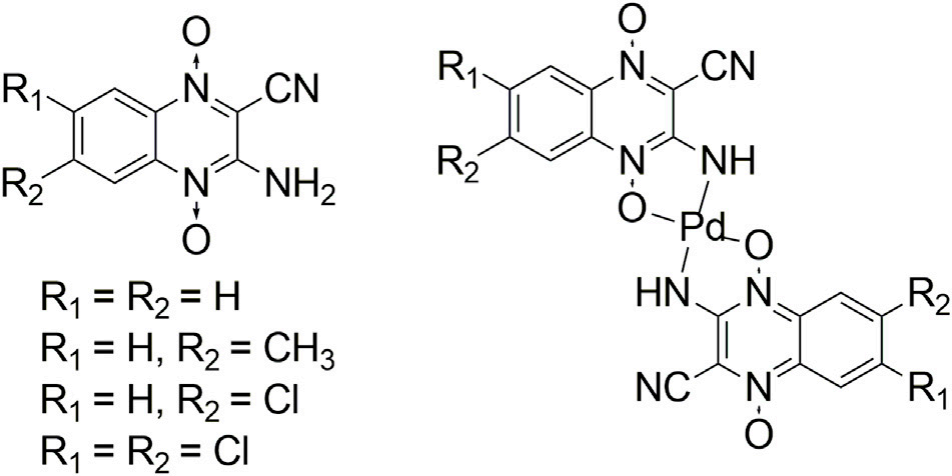
Trypanocidal 3-aminoquinoxaline-2- carbonitrile 1,4-dioxides (quino) and their Pd(II) complexes.

Quinoxalines (3-aminoquinoxaline-2-carbonitrile 1,4-dioxides) are a family of compounds that had shown anti *T. cruzi* activity. Previous QSAR studies had shown that these compounds’ activity was dependent on the electronic characteristics of the substituents as well as on the volume at the 3-amino level of the compounds. However, the low activities displayed by some derivatives could be the result of their low solubilities in the physiological media. In this sense, [Pd(quino)_2_] complexes were obtained in order to improve on one hand, the volume at the 3-amino level (Figure 7) and on the other hand, the bioavailability of the organic ligands. Results showed that the antitrypanosomal activity (epimastigote form, Tulahuen 2 strain) of the quinoxalines was improved upon complexation. The parent ligands having poor trypanosomicidal activity became 20- to 80-times more active upon complexation with palladium (Urquiola et al., 2009; Benítez et al., 2012).

**FIGURE 8:**
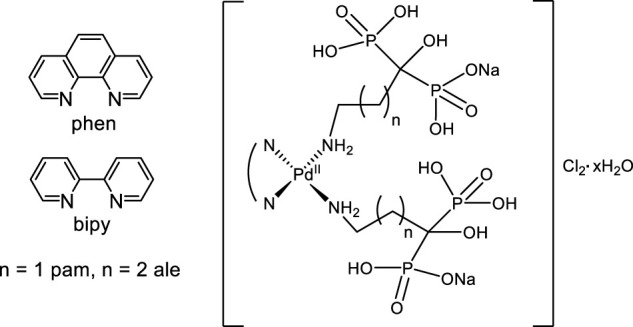
Pamidronate (pam) and alendronate (ale) mixed-ligand Pd(II) compounds.

In order to diminish the costs of developing new drugs, “repositioning” was used as one of the strategies for obtaining new antiparasitic compounds. Biphosphonates are examples of this approach. These compounds are the most prescribed drugs for osteoporosis and other bone diseases. Among bisphosphonates, those containing nitrogen (NBPs) have proved to inhibit farnesyl diphosphate synthase (FPPS) enzyme of the osteoclastic cells as their main mode of action. This enzyme is also present in trypanosomatid parasites. In addition, the specificity of the anti *T. cruzi* action of NBPs could be facilitated by the presence in parasites of specific organelles called acidocalcisomes. Acidocalcisomes are acidic structures involved in the storage and metabolism of phosphorous and calcium in parasites. Their composition is equivalent to the bone mineral so accumulation of bisphosphonates in these organelles would facilitate their antiparasitic action (Gambino and Otero, 2019). Therefore, bisphosphonates were selected as bioactive ligands for our multi-target based approach. In this sense, complexes of the formula [Pd(NBP)_2_(NN)] with NBP = commercial bisphosphonates (alendronate (ale) or pamidronate (pam)) and NN = 1,10 phenanthroline (phen) or 2,2′-bipyridine (bpy) were obtained (Figure 8). The selection of palladium as metal ion and the NN compounds as co-ligands points at DNA as target. In fact, besides the potential covalent interaction of Pd ion with DNA, metal complexes with these planar aromatic ligands could interact with DNA through intercalation between nucleobases. Additionally, parasitic enzymes of the mevalonate pathway (like FPPS) would also be a potential target for these compounds as they are for the NBP ligands.

All the obtained compounds showed an increased anti-*T. cruzi* activity (amastigotes, CL strain) when compared to the free NBP ligands showing only slight signs of unspecific toxicity at high concentrations. In addition, Pd–NBP–phen complexes (IC_50_/3 days = 1.30 and 1.44 µM for ale and pam, respectively) resulted 15 times more active than the corresponding bpy analogues (IC_50_/3 days = 17.4 and 21.4 µM for ale and pam, respectively). However, all the complexes were able to similarly inhibit *T. cruzi* farnesyl diphosphate synthase and solanesyl diphosphate synthase enzymes suggesting that enzymatic inhibition would not be responsible for the observed differences in the biological activity. On the contrary, differences in the anti *T. cruzi* activity could be explained through the interaction of the complexes with DNA. As expected, the nature of the NN ligand determined the complexes’ interaction with DNA. In fact, both Pd–NBP–phen complexes showed a much higher affinity for DNA in the fluorescent ethidium bromide displacement experiments than Pd–NBP–bpy analogues. It should be noted that, for these complexes, a good correlation between the antiparasitic and antitumor activities was observed. Additionally, the compounds were tested for their antitumoral activities. The cytotoxicity of the complexes on MG-63 osteosarcoma cells was dependent on the nature of the NBP as expected but for the A549 lung adenocarcinoma cells, Pd–NBP–phen compounds showed the highest cytotoxicities (Cipriani et al., 2020).

## Ferrocenyl Derivatives

Organometallic compounds, characterized by showing at least one σ metal-carbon bond, offer a promising opportunity for the rational design of novel metal-based drugs. They show a wide structural diversity, their lability can be modulated leading to kinetically stable compounds, and they show adequate lipophilicity which favors their *in vivo* behavior (Allardyce et al., 2005; Hartinger and Dyson, 2009; Noffke et al., 2012; Zhang and Sadler, 2017; Chellan and Sadler, 2020).

**FIGURE 9:**
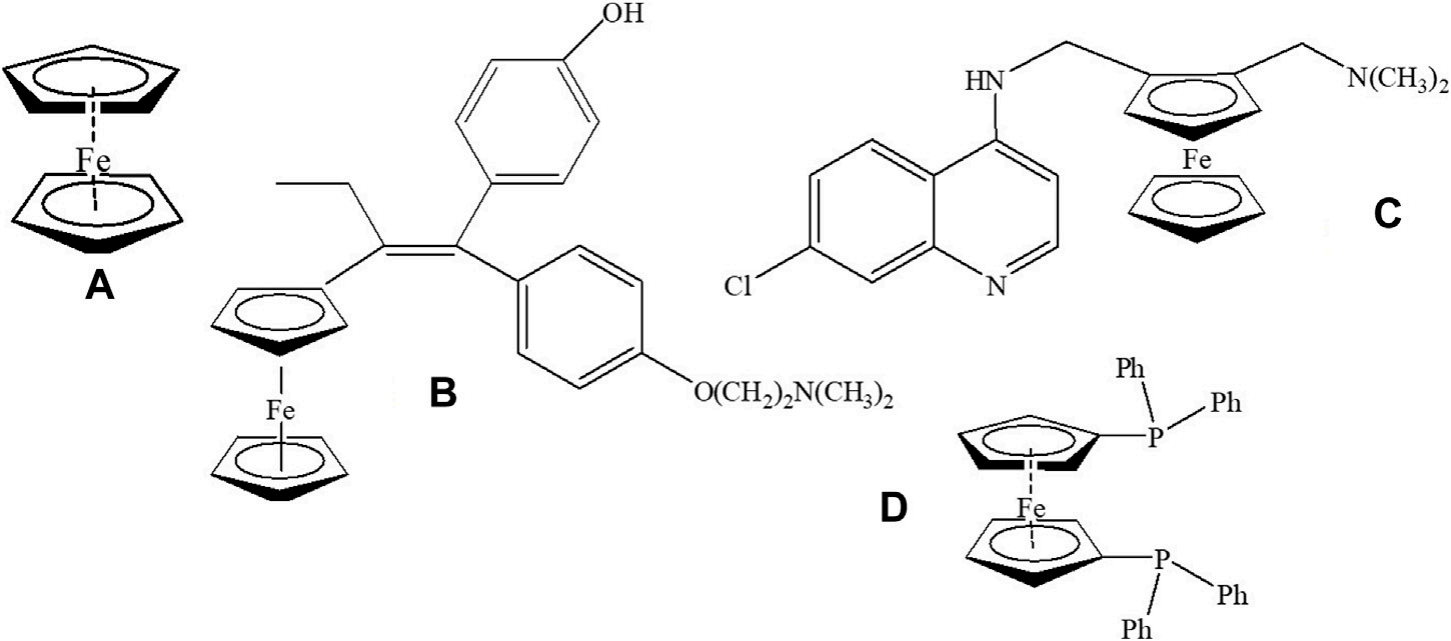
**(A)** ferrocene moiety; **(B)** ferrocifen **(C)** ferroquine **(D)** 1,1'-bis(dipheny1phosphino) ferrocene, dppf.

In particular, the “sandwich type” ferrocene moiety has shown high potentiality in the development of novel organometallic drugs (Figure 9).

For instance, compounds including it, like ferrocifen, an analogue of the antitumoral drug tamoxifen, and ferroquine, an analogue of the antimalarial drug chloroquine, have achieved clinical or preclinical trials (Figure 9). In general, ferrocene derivatives are low cost, they are stable both in air and in solution and they are easy to derivatize. In addition, they are hardly not cytotoxic and have adequate lipophilicity. Furthermore, ferrocene derivatives show improved bioaccumulation when compared to the ionic forms of iron. In addition, ferrocenes are able to undergo one electron oxidation which could catalyze the generations of radicals in a Fenton-like manner, leading to the oxidation of macromolecules. The formation of ROS could be significant for antiparasitic therapy because trypanosomatids have a very primitive system of radical species detoxification (Biot, 2004; Dubar et al., 2008; Biot et al., 2010; Braga and Silva, 2013; Jaouen et al., 2015; Gambino and Otero, 2018).

**FIGURE 10:**
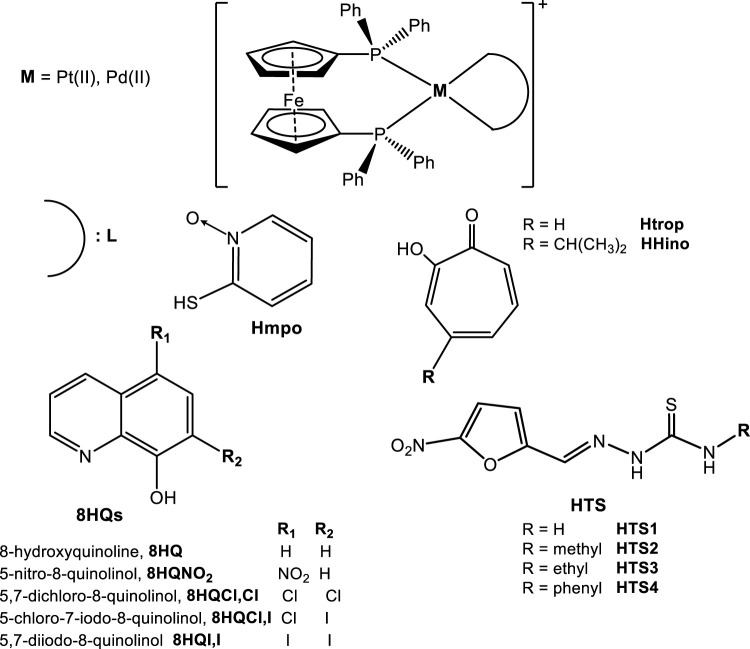
Pd(II) and Pt(II) dppf compounds with selected bioactive ligands L, [M(dppf)(L)](PF_6_).

In order to further address the therapeutic potential of palladium and platinum compounds with the selected bioactive ligands, we followed the rational design strategy previously delineated by including a ferrocene moiety in the new structures (Figure 9). Instead of coupling, as usual, the ferrocene scaffold to an organic skeleton as in ferroquine or ferrocifen, our synthetic strategy was to include the ferrocene fragment as a coligand in the platinum or palladium coordination sphere. The selected coligand, 1,1'-bis(dipheny1phosphino) ferrocene, dppf (Figure 9), acts as bidentate ligand binding to the metal center through the two phosphorus donor atoms and leaving the two extra coordination positions of the metal centre able to coordinate to the selected bioactive bidentate ligand (Figure 10). Accordingly, the twenty-four structurally related ferrocenyl compounds shown in Figure 10 were synthesized and fully characterized in the solid state and in solution and they were evaluated on trypanosomes as well as on mammalian cell models. Their effect on some selected molecular targets was studied and omic studies were performed for the most promising pyridine-2-thiolato-1-oxide (mpo) compounds. In general, the inclusion of the ferrocene moiety led to interesting effects on the biological profile of the compounds (Rodríguez Arce et al., 2015; Mosquillo et al., 2018a; Mosquillo et al., 2018b; Rivas et al., 2018; Rivas et al., 2019; Rodríguez Arce et al., 2019; Mosquillo et al., 2020; Rivas et al., 2021).

**FIGURE 11:**
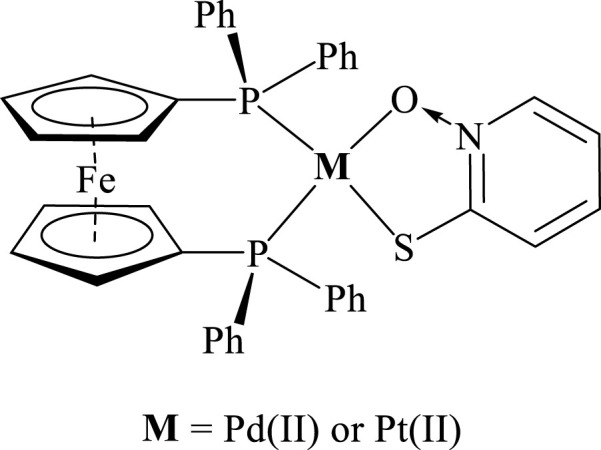
Structure of [M(dppf)(mpo)] (PF_6_) compounds, where M = Pd or Pt, mpo = pyridine-2-thiolato-1-oxide.

At a first stage [M(dppf)(L)](PF_6_) compounds with L = pyridine-2-thiolato-1-oxide (mpo) as bioactive ligand were synthesized and characterized and their biological behavior compared with the previously developed classical coordination compounds [M(mpo)_2_] (Figure 11; Rodríguez Arce et al., 2015). Both ferrocenyl compounds showed IC_50_ values in the nanomolar range on *T. cruzi* epimastigotes (Dm28c strain) as well as low cytotoxicity on VERO epithelial cells (ATCC CCL81) as mammalian cell model, leading to good selectivity towards the parasite (Table 1)**.** The complexes were about 10–20 times more active than the antitrypanosomal drug Nifurtimox (IC_50_/5 days = 6.0 μM) and two- to five-fold more active than mpo sodium salt. Moreover, epimastigotes of CL Brener strain (type VI) resulted more susceptible to the compounds than the type I Dm28c strain (Table 1). It is well known that genetic diversity of the parasite can lead to different susceptibility to drugs.

**TABLE 1:** Activity against trypanosomes of M-dppf-L compounds.

Compound	*T. brucei* IC_50_/24 h/µM	SI^a^	*T. cruzi* IC_50_/µM	SI^a^
Pt-dppf-mpo	—	—	0.28^b^/0.060^c^	18/85 (Vero cells)
Pd-dppf-mpo	—	—	0.64^b^/0.30^c^	39/83 (Vero cells)
Pt-dppf-trop	2.1	18 (J774)	—	—
Pd-dppf-trop	1.3	8 (J774)	—	—
Pt-dppf-hino	4.5	>22	—	—
Pd-dppf-hino	1.2	3	—	—
Pt-dppf-TS1	0.77	>65 (EA.hy926)	3.11^d^	>16 (EA.hy926)
Pd-dppf-TS1	0.9	>55 (EA.hy926)	7.58^d^	>7 (EA.hy926)
Pt-dppf-TS2	0.60	>83 (EA.hy926)	0.79^d^	>63 (EA.hy926)
Pd-dppf-TS2	0.93	>54 (EA.hy926)	1.42^d^	>35 (EA.hy926)
Pt-dppf-TS3	0.52	>56 (EA.hy926)	0.76^d^	>66 (EA.hy926)
Pd-dppf-TS3	0.98	>51 (EA.hy926)	3.6^d^	>14 (EA.hy926)
Pt-dppf-TS4	1.01	>50 (EA.hy926)	1.32^d^	>38 (EA.hy926)
Pd-dppf-TS4	1.56	>32 (EA.hy926)	29.4^d^	>2 (EA.hy926)
Pt-dppf-8HQ	0.3	11.3 (J774)	—	—
Pd-dppf-8HQ	0.9	9.4 (J774)	—	—
Pt-dppf-8HQNO_2_	0.93	27.7 (J774)	—	—
Pd-dppf-8HQNO_2_	0.33	102.4 (J774)	—	—
Pt-dppf-8HQCl,Cl	0.22	15.5 (J774)	—	—
Pd-dppf-8HQCl,Cl	4.5	4.4 (J774)	—	—
Pt-dppf-8HQCl,I	0.14	47.8 (J774)	—	—
Pd-dppf-8HQCl,I	4.8	6.3 (J774)	—	—
Pt-dppf-8HQI,I	0.22	29.1 (J774)	—	—
Pd-dppf-8HQI,I	7	7.2 (J774)	—	—
Nifurtimox	15	10 (J774)	6 (Dm28c epimastigotes)	—
			2.8 (CL Brener epimastigotes)	
			20 (Dm28c trypomastigotes)	

a SI = IC_50_ mammalian cells/IC_50_ parasite.

b Dm28c strain epimastigotes, 24 h incubation.

c CL Brener strain epimastigotes, 24 h incubation.

d Dm28c strain tripomastigotes, 24 h incubation; EA.hy926 endothelial cell line: permanent human cell line derived by fusing human umbilical vein endothelial cells-HUVEC with human lung cells-A549; VERO cells, VERO kidney epithelial cells from African green monkey (ATCC CCL81); J774, J774 murine macrophages.

The compounds induce necrosis after 24 h of parasite incubation. Both complexes also affected the trypomastigote infection process as well as the number of amastigotes per cell. As expected, these dppf compounds showed lower unspecific cytotoxicity on mammalian cells than the [M(mpo)_2_] compounds (Table 1; Mosquillo et al., 2018a; Mosquillo et al., 2018b).

Molecular docking studies conducted on a model structure of the *T. cruzi* NADH fumarate reductase (TcFR) together with experimental *in vitro* studies on *T. cruzi* protein extracts demonstrated the inhibitory effect of the compounds on TcFR. As a consequence of palladium and platinum complexation, an increase in the inhibitory effect in respect to the free mpo was observed. Interestingly, the Pt compound had both the highest inhibition values and the highest activity against *T. cruzi* suggesting that TcFR could be involved in the mode of action of the compounds. Additionally, theoretical calculations confirmed that both compounds could be able to undergo oxidation at the ferrocene moiety which could aid to the biological activity. The generation of radical species inside the parasite cells by the action of M-dppf-mpo compounds was confirmed through EPR experiments (unpublished results) (Gambino and Otero, 2018).

These compounds resulted highly promising deserving further studies. The identification of molecular targets and the understanding of the mode of action of drug candidates are essential data for their clinical development. This knowledge is commonly elusive for metal-based drugs due to complicated mechanisms of action involving the interaction of metal compounds with multiple targets and biomolecules. Inorganic medicinal chemists have traditionally tried to identify and characterize, using *in vitro* approaches, molecular targets of a metal-based drug that mainly depend on the nature of the organic ligands and the metal center. Omic studies are relevant tools for uncovering the whole mechanism of action of metal-based drugs. Up and down regulated cellular proteins due to effects of drug and molecular targets interactions, identified and quantified by proteomics, together with cell uptake and subcellular distribution, quantified by metallomics, and changes in gene expression, determined by transcriptomics, allow to get a deeper insight into what occurs in the cells after administering a metallodrug. The knowledge achieved is a useful income for the rational design of novel metallodrugs (Wang et al., 2020).

High-throughput omic studies have proved to be powerful tools for going further into the basic biology of parasites like *T. cruzi* and also for the validation of drug targets (Atwood et al., 2005; Chávez et al., 2017; García-Huertas et al., 2017; Van den Kerkhof et al., 2020). Although these studies have been also used to explore organic drugs action in kinetoplastid parasites, similar studies for metal-based drugs were not reported until part of our group recently performed a high throughput omic study on *T. cruzi* for the two analogous Pt and Pd organometallic hit compounds [M^II^(dppf)(mpo)](PF_6_) (Mosquillo et al., 2018a; Mosquillo et al., 2018b; Mosquillo et al., 2020).

Metallomic studies showed a high Pt and Pd uptake by parasite epimastigotes. For the same dose a higher nanomolar uptake per 10^6^ parasites was determined for Pt-dppf-mpo than for Pd-dppf-mpo. A similar pattern of metal distribution among the four analyzed macromolecules fractions (DNA, RNA, soluble proteins and insoluble proteins including membrane lipids) was found, with a preferential association to DNA. It is well known that metal complexes often could suffer reactions in biological media. Therefore, to evaluate if the M-dppf-mpo compounds are taken up intact by the parasites, the M (Pd or Pt) and Fe (present in the dppf moiety) levels were quantified in the selected parasite fractions at the same time. A 1:1 stoichiometric relationship between Fe and M in each fraction was obtained which would be consistent with the presence of the intact M-dppf-mpo species bound to the selected macromolecules (Mosquillo et al., 2018a; Mosquillo et al., 2018b). Proteomic and transcriptomic analyses allowed to identify differentially expressed transcripts and proteins in the treated parasites. The number of differentially expressed proteins was 342 for Pd-dppf-mpo and 411 for Pt-dppf-mpo. In addition, the number of soluble and insoluble proteins modified after treatment with both compounds was similar. The effect of Pd-dppf-mpo treatment showed more modulated transcripts (2,327 of 10,785 identified transcripts) than Pt-dppf-mpo treatment (201 of 10,773 identified transcripts). These results suggest that the mechanism of action for Pd-dppf-mpo is at the transcriptome level. Differentially expressed transcripts were functionally categorized which allowed to identify the cellular processes and pathways that were affected by the treatment with the compounds. Transcripts involved in DNA binding, protein metabolism, transmembrane transport (ABC transporters related with *T. cruzi* response and resistance to drugs, among others), oxidative defense, and the biosynthesis of ergosterol pathways were found to be modulated by the presence of the compounds. The whole set of data obtained allowed to suppose that the antitrypanosomal mechanism of action of Pd-dppf-mpo and Pt-dppf-mpo is multimodal. Interestingly, significant biological differences were assessed for both structurally analogous compounds showing the significance of the nature of the metal center on the biological behavior. In particular, metallomic studies allowed to assess that the Pt(II) compound showed higher cellular uptake than the Pd one. In addition, proteomic and transcriptomic studies showed different biological effects of both chemically analogous compounds. Both metals belong to the same group of the periodic table allowing to expect many chemical and physicochemical similarities between their compounds. Nevertheless, differences are expected based on the differential lability of the metal centers. The omic work performed gives details about the biological implications emerging of these chemical differences (Mosquillo et al., 2020).

As previously discussed, NADH-fumarate reductase, a specific parasite enzyme absent in the host, had been previously identified as a potential target for both M-dppf-mpo compounds (Mosquillo et al., 2018a). Although proteomic analysis agrees with this previous finding showing that, the amount of enzyme is modified in the treated parasites respect to untreated control parasites, omic studies unveiled, in addition, several down and up regulated proteins and a wide range of pathways affected by the compounds. Another remarkable point is that *in vitro* target identification should be in agreement with observed cellular uptake and subcellular localization. In the case of the M-dppf-mpo compounds, DNA was not considered for the *in vitro* target identification as an important biological target to be tested but metallomics showed a high accumulation of the complexes in the parasite DNA fraction. Globally, this study constitutes the first omic contribution to unravel the whole scenario of effects involved in the mechanism of action of potential metal-based drugs for the treatment of Chagas disease.

Following the promising results obtained with the M-dppf-mpo compounds and in an attempt to get broad spectrum Pd and Pt dppf compounds that could affect both *T. brucei* and *T. cruzi* parasites, four 5-nitrofuryl containing thiosemicarbazones (HTS) were coordinated as bioactive bidentate ligands to the {M-dppf} centers leading to eight new heterobimetallic [M^II^(TS)(dppf)](PF_6_) Pt(II) or Pd(II) compounds (Figure 10). IC_50_ values for most compounds were in the low micromolar or submicromolar range against both parasites, having the platinum compounds higher activities than the corresponding palladium ones (Table 1). Their activities were significantly higher than those of the free thiosemicarbazone ligands, showing a 3- to 24-fold increase for *T. cruzi* and up to 99-fold increase for *T. brucei*. The presence of the organometallic dppf co-ligand also seems to be responsible for a lower toxicity on mammalian cells and higher selectivity towards both parasites when compared to the free thiosemicarbazone compounds. In addition, new compounds showed higher activity and selectivity than the several groups of Pd and Pt classical coordination complexes with 5-nitrofuryl thiosemicarbazones previously described in this review.

The M-dppf-TS compounds resulted up to 26 times more active on *T. cruzi* than Nifurtimox (IC_50_/24 h = 20 μM) and up to 30 times (IC_50_/24 h = 15 μM) on *T. brucei*. These Pd and Pt compounds demonstrated to affect the redox metabolism of *T. cruzi* seeming to retain the mechanism of anti-*T. cruzi* action of the free ligands previously described in this review. However, no correlation between oxygen uptake and the generation of free oxygen radical species in the parasite, and the anti-*T. cruzi* activity was observed. Additionally, these compounds demonstrated to interact with DNA. Fluorescence results showed that ethidium bromide (EB) was displaced from the {DNA–EB} adduct as a result of the interaction of the Pd and Pt complexes with the biomolecule. This effect could be related to an intercalative-like mode of interaction or to the generation of DNA conformational changes causing the disruption of EB binding (Log *K*_SV_ 4.3–5.0, with *K*_SV_ = Stern Volmer quenching constant). Obtained *K*_SV_ values could be related to a high affinity of the compounds for DNA. However, no correlation between the interaction with DNA and the biological activity was observed, discarding this biomolecule as a main target.

Zebrafish (*Danio rerio*) is employed as a toxicological model, among others, for evaluating *in vivo* toxicity in drug development. The most active and selective compound of the new series, [Pt(dppf)(TS3)] (PF_6_), showed no *in vivo* toxicity in zebrafish embryos. All embryos were alive in the 1–100 μM concentration range examined, and no apparent toxicity was observed after 48 h of treatment (Rodríguez Arce et al., 2019).

Based on the very nice results obtained for these complexes on both, *T. cruzi* and *T. brucei*, other ferrocenyl compounds, [M(dppf)(L)](PF_6_), with M=Pd(II) or Pt(II) and HL=tropolone (HTrop) or hinokitiol (HHino), were obtained and evaluated against the bloodstream form of *T. brucei* and *L. infantum* amastigotes (Figure 10; Rivas et al., 2018). Tropolones and their derivatives were selected as bioactive ligands because they are considered lead-like natural products. The tropolone moiety shows high possibilities for derivatization including improvements in the metal binding abilities. Tropolone, hinokitiol and their derivatives as well as the metal complexes with these compounds as ligands have shown various biological activities, for example antimicrobial one (Ononye et al., 2013; Saniewski et al., 2014; El Hachlafi et al., 2021).

The obtained complexes showed IC_50_/24 h values in the range 1.2–4.5 μM against *T. brucei* together with a significant increase of the activity against this parasite with respect to the free ligands (Table 1). In addition, obtained heterobimetallic compounds showed higher selectivity indexes towards the parasite than the free ligands. Platinum complexes were more selective than palladium ones. Moreover, coordination of the bioactive ligands to the {M-dppf} moiety also led to a slight increase of the anti-leishmanial potency. Studies performed to unravel the mechanism of action of the compounds indicated that no effect on the thiol-redox homeostasis of the parasites is produced by the complexes’ action. On the other hand, fluorescence measurements of displacement of ethidium bromide from the adduct {DNA-EB} showed that DNA could be a probable, but not main, target of these compounds.

Later on, the series of structurally related Pd and Pt dppf compounds was expanded with ten new compounds that include five 8-hydroxyquinoline derivatives (8HQs) as bioactive bidentate co-ligands (Figure 10; Rivas et al., 2019; Rivas et al., 2021). These compounds showed IC_50_ values against bloodstream *T. brucei* form, in the submicromolar or micromolar range (IC_50_/24 h: Pt compounds 0.14–0.93 μM; Pd compounds 0.33–1.2 μM). In addition, they displayed good to very good selectivity towards the parasite (SI: Pt compounds 11–48; Pd compounds 4–102) with respect to murine macrophages (cell line J774) (Table 1). In most cases, an increase of the activity (11- to 41-fold) was observed as a consequence of coordination of the bioactive 8HQs to the {Pt-dppf} moiety. Only part of the Pd compounds were more active than the corresponding 8HQ ligands and most of the Pd compounds were less active than their Pt analogues. It should be stated that, the palladium compounds were 2- to 45-fold more potent than the drug Nifurtimox while platinum complexes resulted 16- to 107-fold more potent than the same reference drug. All the complexes interacted with DNA (Pt compounds Log *K*_SV_ 3.3–3.9; Pd compounds Log *K*_SV_ 3.8–4.6) but the Pd compounds show higher Log *K*_SV_ values than the Pt analogues. In addition, the most active Pt ones induced reactive oxygen species (ROS) formation in tumor cells. Results suggest that the mechanism of action for these complexes against *T. brucei* may be mediated by interaction with DNA and additionally oxidative stress for the Pt compounds.

An exploratory pre-clinical therapeutic efficacy study was performed in an acute murine model for Human African Trypanosomiasis (HAT) for the most promising Pt compound, Pt-dppf-8HQ Cl,I, (IC_50_/24 h = 0.14 μM, SI = 48) using mice infected with a bioluminescent cell line of *T. brucei* that allows *in vivo* mice imaging. Although preliminary, the *in vivo* study showed that the assayed compound does not show acute toxicity to animals. In addition, results suggested that although the compound exerts an anti-proliferative activity that prolongs animal survival, it does not exert a curative effect (Rivas et al., 2021).

Having developed a long series of twenty-two structurally related M-dppf-L compounds with different bioactive ligands L (Figure 10) that had been evaluated against bloodstream *T. brucei* and with the aim of understanding and assessing the main structural parameters that determine the anti-*T. brucei* activity, a quantitative structure–activity relationships (QSAR) study was performed (Rivas et al., 2021). For this study, the dependent variable was the log_10_ of the IC_50_ values on *T. brucei*. As independent variables, different physicochemical characteristics including lipophilic, electronic, and steric/topological properties were considered (Hansch et al., 1963). Lipophilicity was indirectly determined by a reverse phase TLC method adequate for non-soluble in water compounds that led to experimental R_f_ and calculated R_M_ values. As electronic parameter a signal of the ^1^H NMR spectra of the studied compounds was selected. Owing to the chemical variability of the studied compounds, i.e. palladium or platinum and three different families of ligands, the cyclopentadienyl-moiety was selected as common feature to determine the contribution of the compounds’ electronic effect on the measured anti *T. brucei* activities. The displacement δ of the ^1^H NMR signals of the protons of the cyclopentadienyl-framework resulted indicative of the coordination and the nature of compound. Consequently, Δδ defined as the largest difference between δ of cyclopentadienyl-framework protons in the complexes and δ of cyclopentadienyl-framework protons without coordination was used as electronic descriptor. Additionally, an indicator variable, IV_Pd_, was defined that adopts value 1 for palladium compounds or 0 for platinum complexes. According to the QSAR study, ligands with electron withdrawing substituents and with high lipophilicity and having platinum as central atom would result in complexes with increased anti *T. brucei* activity. Among all these descriptors, the electronic properties and the nature of the metal ion were the most relevant ones. QSAR studies are relevant to guide the rational design of further bioactive compounds. However, they are not common in inorganic medicinal chemistry due to the need of having numerous structurally related compounds to perform them (Rivas et al., 2021).

## Concluding Remarks

The development of metal-based compounds for the treatment of diseases caused by trypanosomatid parasites has evolved from rather isolated serendipitous efforts to a more rational and systematic strategy. In this sense, the development of palladium and platinum compounds described in this review constitute an example of this rational phenotypic approach. In fact, from the selection of the metal centers and the bioactive ligands to the inclusion of different co-ligands, the design was based on both chemical and biological arguments. The establishment of structure-activity relationships and the deep insight into the molecular modes of action of the metallic compounds also aided to redesign new compounds with improved pharmacological properties. In this sense, the development of structurally related series of compounds has let us perform quantitative structure activity relationship (QSAR) studies that are not common in Medicinal Inorganic Chemistry.

On the other hand, all the data previously discussed clearly show that the strategy of combining, in a single molecule, palladium or platinum with ligands bearing activity against parasites produce, in most cases, an enhancement of the activity of the ligand and/or a reduction in toxicity. In addition, it was demonstrated that this approach could lead to multifunctional compounds generating single chemical entities that can act simultaneously on multiple targets. In this sense, the omic approach, also new for metal-based antiparasitic drug development, allowed us to go further into the study of the whole mechanism of action of the prospective antiparasitic agents.

In the process of rational design of palladium and platinum complexes bearing antiparasitic activity, the selection of dppf as co-ligand deserves to be highlighted. The inclusion of a bioactive ligand in the {M-dppf} moiety gave the most promising results as most developed ferrocenyl derivatives not only showed low IC_50_ values in both *T. cruzi* and *T. brucei* but also excellent selectivity index values. In particular, the hypothesis of developing broad spectrum drugs that affect more than one trypanosomatid parasite, based on the discovery of common genomic features for *T. cruzi*, *T. brucei* and *L. major*, was verified in the case of the 5-nitrofurylthiosemicarbazone M-dppf-L complexes. These compounds showed IC_50_ values in the submicromolar or micromolar range on both, *T. cruzi* and *T. brucei*. Some of these compounds were good candidates for *in vivo* studies. Currently, our group is working on the development of delivery systems based on different nano-systems with the aim of avoiding or diminishing usual toxicity and solubility problems of metal-based drugs and to improve bioavailability.
